# Cotyledonoid dissecting leiomyoma of the uterus: a case report and review of the literature

**DOI:** 10.1186/s13256-023-04271-8

**Published:** 2023-12-16

**Authors:** Mahboobeh Chahkandi, Marzieh Ataei, Amir Reza Bina, Farnaz Mozayani, Ali Fanoodi

**Affiliations:** 1 Department of Pathology, School of Medicine, Birjand University of Medical Sciences, Birjand, Iran; 2 Department of Obstetrics and Gynecology, Clinical Research Development Unit, Vali-e-Asr Hospital, Birjand University of Medical Sciences, Birjand, Iran; 3 Student Research Committee, School of Medicine, Birjand University of Medical Sciences, Birjand, Iran; 4 Cellular and Molecular Research Center, Birjand University of Medical Sciences, Birjand, Iran

**Keywords:** Cotyledonoid dissecting leiomyoma, Case report, Uterus, Leiomyoma

## Abstract

**Background:**

Cotyledonoid dissecting leiomyoma, also named Sternberg tumor, is a rare variant of uterine leiomyoma. The tumor is benign, but the appearance and growth pattern are unusual and alarming. In this article, we report a case of cotyledonoid dissecting leiomyoma in a 55-year-old woman as well as review relevant literature.

**Case presentation:**

We report a case of cotyledonoid dissecting leiomyoma in a 55-year-old Iranian woman who presented with vaginal bleeding 4 months after menopause. Ultrasound showed two heterogeneous hypoechoic masses on the uterine fundus. Total abdominal hysterectomy and bilateral salpingo-oophorectomy were performed for the patient. Macroscopically, a large heterogeneous intramural mass (140 mm × 120 mm × 120 mm) with a grape-like exophytic mass on the fundus was observed. Her health status was good after surgery, and the patient was discharged from the hospital after 2 days. In a 1-year follow-up period, no recurrence or any other related complications were found.

**Conclusion:**

It is important to recognize this rare variant of leiomyoma to prevent aggressive and inappropriate overdiagnosis and overtreatment. It is suggested to try to use frozen sections for better diagnosis and to preserve fertility in young women suffering from this lesion.

## Background

Uterine leiomyomas have received great attention in recent years; however, the exact pathogenesis of uterine leiomyoma growth is not completely uncovered. Several agents such as growth factors, cytokines, chemokines, estrogen, progesterone, and human chorionic gonadotropin (HCG) are suggested to be involved in the growth of these tumors [1, 2]. Uterine leiomyomas might be found during an ultrasound examination; however, sometimes patients refer with abdominal pain and discomfort or pregnancy-related complications, including placental abruption, retained placenta, preterm labor, or postpartum hemorrhage [1, 3].

Cotyledonoid dissecting leiomyoma, also known as Sternberg tumor, is a benign variant of leiomyoma with an unusual macroscopic appearance. It was reported for the first time in 1996 by Roth *et al*. [4]. Presently, only a few cases have been reported worldwide, in such a way that Jamal *et al*. call this tumor an uncommon form of a common disease [5]. The lesion usually shows an exophytic mass-like gross appearance of placental tissue and extends into the myometrium with dissection of myometrial fibers [6–8]. This gross appearance may mimic uterine malignancy [8, 9].

We report a case of cotyledonoid dissecting leiomyoma in a 55-year-old postmenopausal woman who presented with postmenopausal vaginal bleeding and underwent total abdominal hysterectomy along with bilateral salpingo-oophorectomy. This case report was written based on the reporting checklist for case report guidelines (CARE guidelines) [10], and written informed consent was obtained from the patient for publication of this case report and any accompanying images. Moreover, this study was approved by Birjand University of Medical Science’s Research Ethics Committee (approval ID: IR.BUMS.REC.1401.119).

## Case presentation

A 55-year-old Iranian woman with a history of two pregnancies and two deliveries presented with postmenopausal vaginal bleeding, which began 4 months after menopause and lasted for 10 days. Laboratory tests showed moderate normochromic normocytic anemia (hemoglobin 9.8 g/dL, Mean Corpuscular Volume (MCV) 85 fl, Mean Corpuscular Hemoglobin (MCH) 27 pg, and Mean Corpuscular Hemoglobin Concentration (MCHC) 31.6 g/dL). On pelvic physical examination, a mass in the uterus was detected. The transabdominal ultrasound scan demonstrated two solid heterogeneous hypoechoic masses (141 mm × 110 mm and 81 mm × 61 mm) in the myometrium of the uterine fundus. No cystic lesion or mass was found in the adnexa. Hence, for evaluation of malignancy, the patient underwent an endometrial pipelle biopsy, which was normal. Finally, the patient underwent a total abdominal hysterectomy and bilateral salpingo-oophorectomy, and macroscopic and microscopic evaluations were performed.

### Pathologic findings

#### Macroscopic

**Fig. 1:**
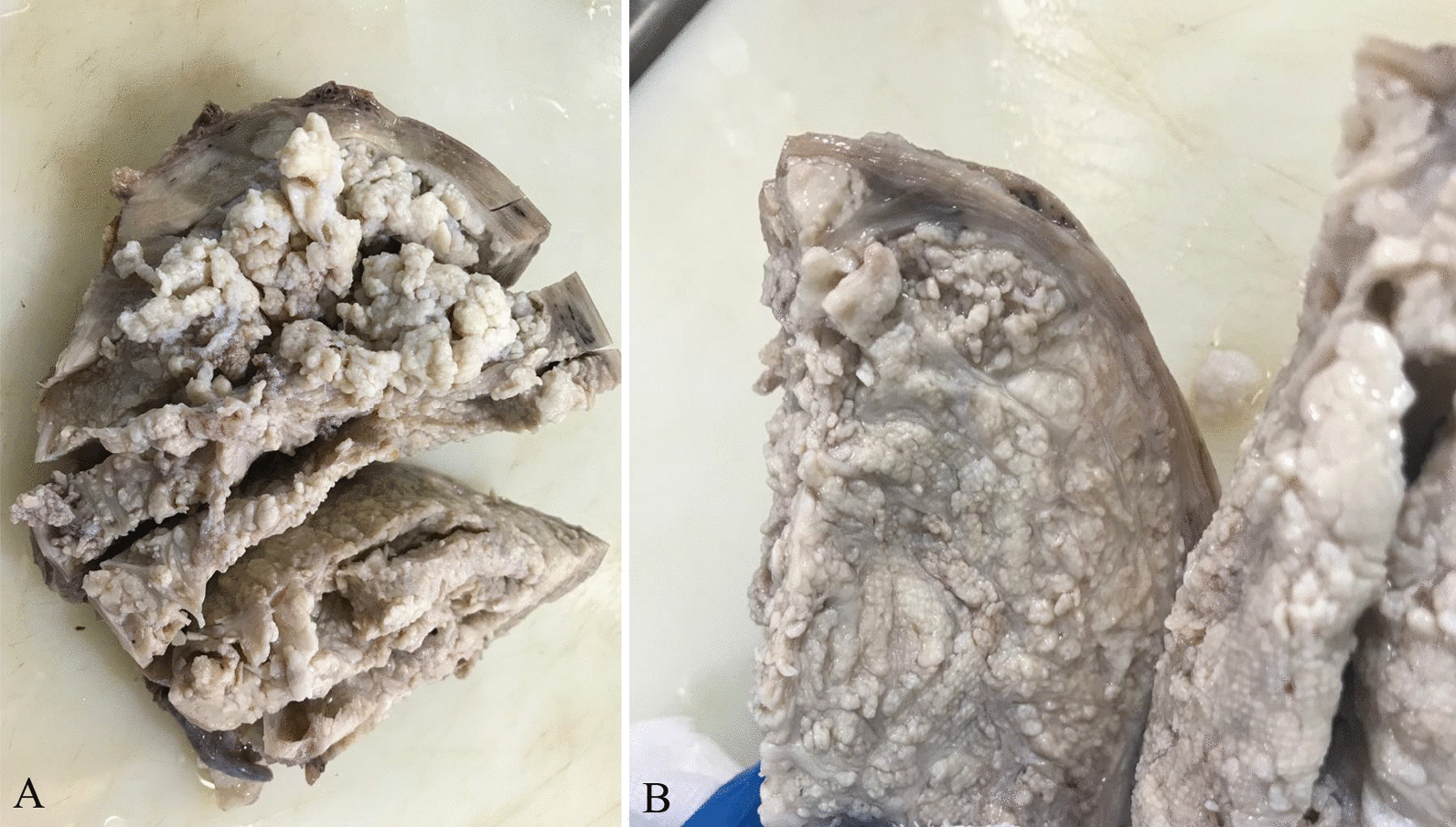
**A** Gross image of cotyledonoid dissecting leiomyoma, giving a placental appearance. **B** Cut section with multiple tan-white nodules. Variable-sized tan-white nodules dissect the myometrium to the serosal surface and make grape-like projections on the serosal surface of the uterine fundus

One tumoral tissue (140 mm × 120 mm × 120 mm) in the uterine fundus was found, originating from the myometrium, compressing the endometrial cavity. The tumor (measuring 35 mm × 30 mm × 30 mm) showed a multinodular appearance, which dissects the myometrium to the serosal surface and makes grape-like projections on the serosal surface of the uterine fundus (Fig. 1).

The boundary between the tumor and the myometrium was unclear. No necrosis was found in the tumor. One polypoid lesion (15 mm × 10 mm × 20 mm) in the endometrial cavity was also seen. Two separated small intramural leiomyomas (15 and 10 mm) were seen in the left uterus wall. No pathologic findings were seen in the bilateral adnexa.

#### Histologic

**Fig. 2:**
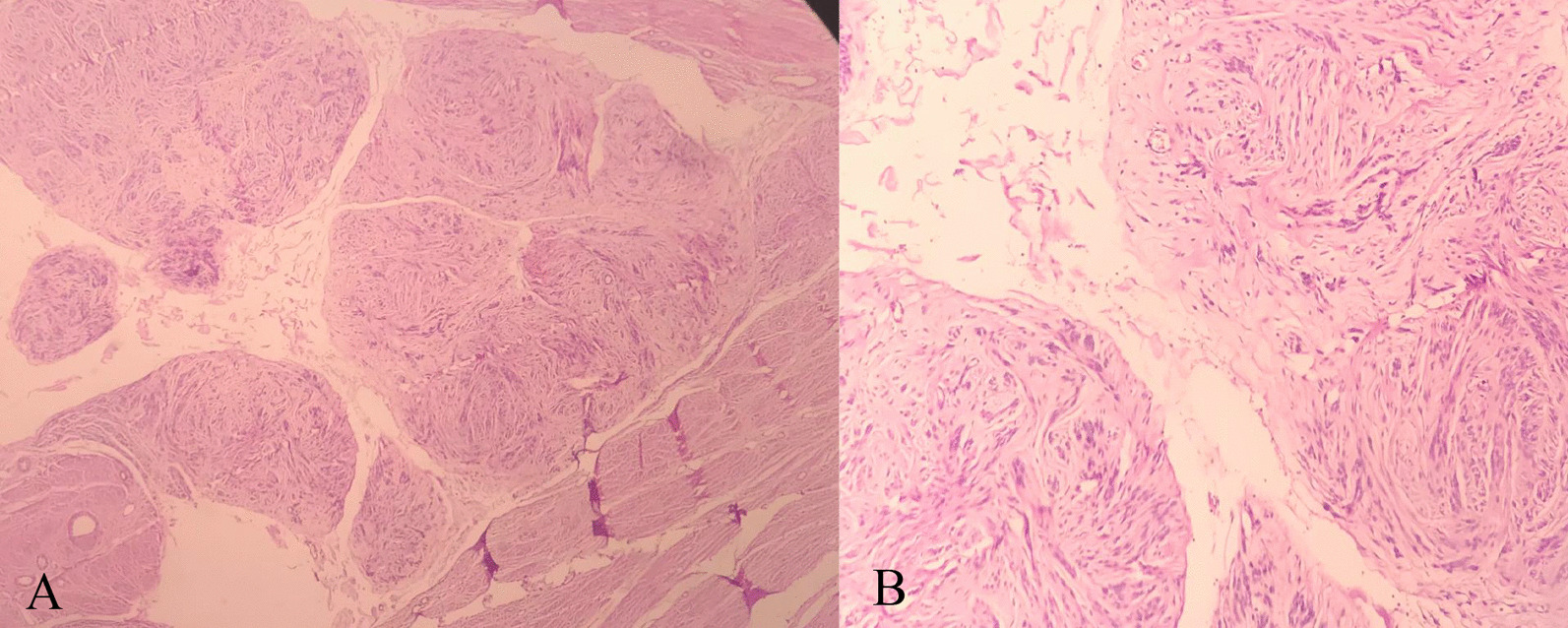
Microscopic images of dissecting nodules [hematoxylin and eosin (H&E) stain, magnification size: **A** ×100, **B** ×400]. The nodules are composed of benign-looking spindle cells without atypia, necrosis, or increased mitosis

**Fig. 3:**
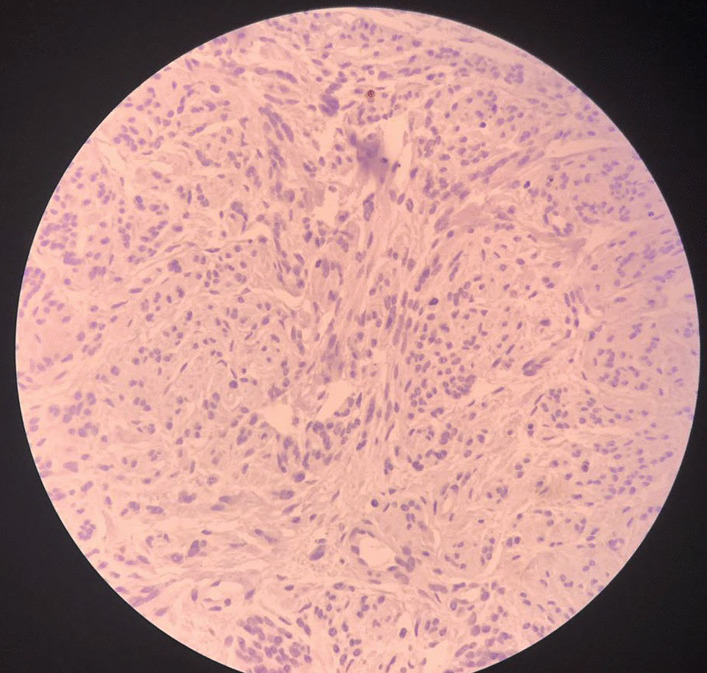
Immunohistochemical staining of cotyledonoid dissecting leiomyoma cells. K_i_-67 staining slides show low mitotic activity (K_i_-67 index < 5%)

The tumor showed multiple nodules composed of smooth muscle fibers arranged in fascicular and whorling structures. Cells showed eosinophilic cytoplasm and bland-looking plump nuclei. A significant stromal edema was seen. No necrosis was noted in the tumor. An intravascular growth was absent (Fig. 2). Mitotic activity was low (K_i_-67 index < 5%) (Fig. 3). According to the above description, we came into the conclusion that the tumor was a cotyledonoid dissecting leiomyoma. An additional pathologic finding, in this case, was an endometrial polyp.

It should be noted that in 1-year follow-up period, no recurrence or any other related complications were found in the patient.

## Discussion

Leiomyoma is the most common benign neoplasm of the female genital system. Cotyledonoid dissecting leiomyoma is a rare variant of leiomyoma with unusual macroscopic appearance, which may mimic malignancy due to its growth pattern in the uterus wall. This tumor was first reported in 1996 by Roth *et al*. [4]. To the best of our knowledge, this is the first report of cotyledonoid dissecting leiomyoma from Iran.

The age range of reported cases was 23–73 years [11]. Clinical presentations include abnormal uterine bleeding, pelvic mass, constipation, bloating, and weight gain. However, the most common presentation is abnormal uterine bleeding, which was found in our case [11].

Cotyledonoid dissecting leiomyoma has some variants:

1. Some reported cases of cotyledonoid dissecting leiomyoma had atypical cells; however, this microscopic finding cannot be diagnostic for malignancy without other criteria [12].
2. A new form of cotyledonoid dissecting leiomyoma named “cotyledonoid hydropic intravascular leiomyomatosis” is also described [13].
3. Another variant called “cotyledonoid leiomyoma” was described by Roth and Reed, which is similar to cotyledonoid dissecting leiomyoma, but lacks an intramural component [14].
4. Another author also described a variant called “intramural dissecting leiomyoma,” which lacks extrauterine placental-like component [15].

Disorganized smooth muscle fascicles as well as marked hydropic degeneration and extensive vascularity are the main key factors to diagnose cotyledonoid dissecting leiomyoma [16]. Cotyledonoid dissecting leiomyoma is a benign tumor, but its appearance is challenging for gynecologists, radiologists, and pathologists. Due to the unusual gross appearance of the tumor, one of the most important differential diagnoses is leiomyosarcoma. Classification of malignant smooth muscle tumors according to the study of Kempson and Hendrickson is based on tumor coagulation necrosis, mitotic activity (K_i_-67 index), and cellular atypia [17]. According to the microscopic findings of our case (no necrosis, no cellular atypia, and low mitotic activity), leiomyosarcoma was excluded.

Table 1 provides a review of some cases of cotyledonoid dissecting leiomyoma with their ages, clinical presentations, tumor size, and tumor location. Despite the macroscopic and microscopic unusual appearance of cotyledonoid dissecting leiomyoma, no malignant behavior has been reported.

**Table 1:** A review of some cases of cotyledonoid dissecting leiomyoma

References		Age	Clinical presentation	Tumor size (maximum dimension, mm)	Tumor location
1	David *et al*. [18]	65	Abnormal uterine bleeding	150	Uterine fundus and cervix
		48	Uterine prolapse	120	Uterine fundus
2	Roth *et al*. [4]	39	Pelvic mass	103	Uterine horns
		41	Abnormal uterine bleeding	100	Uterine horns
		23	Pelvic mass	250	Uterine horns
		Unknown	Pelvic mass	240	Uterine horns
3	Brand *et al*. [19]	24	Abdominal mass	NA	Uterine fundus
4	Roth and Reed [14]	46	Pelvic mass	340	Uterine horns
5	Kim *et al*. [20]	26	Incidental	120	Posterior uterine wall
6	Cheuk *et al*. [21]	55	Abnormal uterine bleeding	100	Uterine horns
7	Stewart *et al*. [22]	58	Abdominopelvic mass	164	Uterine fundus
8	Jordan *et al*. [13]	46	Right adnexal mass	220	Uterine with extrauterine invasion (All cases)
		46	Pelvic mass	200	NA
		46	Pelvic mass	100	NA
		46	Pelvic mass	180	NA
		36	Abnormal uterine bleeding	130	NA
		34	Uterine mass, infertility	180	NA
9	Saeed *et al*. [23]	27	Pelvic mass	410	Uterine fundus
10	Maimoon *et al*. [24]	40	Urinary retention	100	Uterine isthmus
11	Shelekhova *et al*. [25]	73	Uterine mass	80	Uterine fundus
12	Gurbuz *et al*. [26]	67	Incidental	100	Uterine horns
13	Weissferdt *et al*. [27]	52	Abnormal uterine bleeding	62	Uterine fundus
14	Raga *et al*. [28]	33	Abdominal pain	60	Lateral part of uterus
15	Driss *et al*. [29]	47	Pelvic mass	250	Uterine with extrauterine invasion
16	Preda *et al*. [30]	41	Uterine mass	90	Left and posterior uterine wall
17	Fukunaga *et al*. [31]	56	Constipation	300	Posterior uterine wall
		47	Abdominal pain	260	Posterior uterine wall
		36	Abnormal uterine bleeding	40	Posterior uterine wall
		35	Abdominal pain	180	Lateral uterine wall
18	Gezginç *et al*. [32]	57	Pelvic pain	25, 45	Intrauterine, lateral uterine wall
19	Agarwal *et al*. [33]	52	Abnormal uterine bleeding	100	Uterine horns
20	Ersöz *et al*. [16]	51	Abnormal uterine bleeding	85	Subserosal
21	Roth *et al*. [34]	33	Abnormal uterine bleeding	65, 135	Posterior uterine wall
22	Tanaka *et al*. [35]	36	Incidental	100	Posterior and lateral uterine wall
23	Onu *et al*. [36]	50	Incidental	100	Uterine fundus
24	Kim *et al*. [37]	43	Abdominal mass	130	Uterine with extrauterine invasion
25	Blake *et al*. [38]	56	Abnormal uterine bleeding	300	Uterine with extrauterine invasion
26	Shimizu *et al*. [39]	40	Abnormal uterine bleeding	100	Posterior uterine wall
27	Xu *et al*. [8]	55	Pelvic mass	60	Posterior uterine wall
		43	Pelvic mass	30	Body of uterus
		37	Pelvic mass	300	Periuterine
		48	Lower abdominal pain	67	Right wall of uterus
28	Lenz *et al*. [40]	64	Pelvalgia and loss of renal function of the right kidney		Right edge of the uterine wall, the right parametrium, distal part of the right ureter, and the right and the partial wall of the cranial bladder
29	Rocha *et al*. (41)	38	Menorrhagia and abdominal pain	250	Uterine isthmus

*NA* not available

## Conclusion

Although the microscopic or macroscopic appearance of cotyledonoid dissecting leiomyoma might be malignant, no recurrence or aggressive behavior of this tumor has been reported until now. Therefore, the gynecologists, pathologists, and radiologists should be aware enough to recognize this rare variant of leiomyoma to prevent overtreatment. Although the gold standard treatment of this tumor in postmenopausal women is total abdominal hysterectomy and bilateral salpingo-oophorectomy, it is important to preserve fertility in young women who suffer from this lesion. Therefore, it is suggested to try to use frozen sections for better diagnosis.

## Data Availability

Not applicable.
